# Electronic structure, reflectivity and X-ray luminescence of MAPbCl_3_ crystal in orthorhombic phase

**DOI:** 10.1038/s41598-025-96694-0

**Published:** 2025-04-15

**Authors:** Volodymyr Kolomiets, Volodymyr Kapustianyk, Mariya Kovalenko, Hans Kraus, Oksana Chukova, Yaroslav Zhydachevskyy, Wagas Zia, Michael Saliba, Vitaliy Mykhaylyk

**Affiliations:** 1 Physics Department, I. Franko National University of Lviv, 8 Kyrylo and Mefodiy str., Lviv, 79005 Ukraine; 2 Department of Physics, University of Oxford, Denys Wilkinson Building, Keble Road, Oxford, OX1 3RH UK; 3 Deutsches Elektronen-Synchrotron DESY, 85 Notkestr., 22607 Hamburg, Germany; 4 Institute of Physics, Polish Academy of Sciences, al. Lotników 32/46, 02-668 Warsaw, Poland; 5 Berdyansk State Pedagogical University, 4 Shmidta str., Berdiansk, 71100 Ukraine; 6 Institute for Photovoltaics, University of Stuttgart, 70569 Stuttgart, Germany; 7 Helmholtz Young Investigator Group FRONTRUNNER, IEK-5 Photovoltaics, Forschungszentrum Jülich, 52425 Jülich, Germany; 8 Diamond Light Source, Harwell Campus, Didcot, OX11 0DE UK

**Keywords:** Condensed-matter physics, Semiconductors, Physics, X-rays

## Abstract

This study provides a comprehensive analysis of the electronic structure, reflectivity, and luminescent spectra of the organic-inorganic, metal-halide MAPbCl_3_ perovskite, which has considerable potential for various optoelectronic applications. Using density functional theory (DFT) calculations, we investigated the electronic structure of MAPbCl_3_ and interpreted the key features of its reflectivity spectra across a wide energy range from 3 to 10 eV. The reflectivity spectra reveal prominent excitonic features at 3.22 eV near the absorption edge and additional optical transitions at higher energies, highlighting the material’s intricate electronic structure. Furthermore, we examined the temperature dependence of radiative decay dynamics under high-energy radiation through X-ray luminescence spectra and decay time measurements. We observe emission from free and bound excitons with an exceptionally short decay time (≤ 1 ns) and significant thermal quenching at low temperatures (100 K) in the 385–430 nm range. These findings underline the importance of continued exploration of optoelectronic properties of the material to enhance its performance in practical applications.

## Introduction

In recent years metal-halide perovskites with the general formula APbX_3_ (A = methylammonium (MA, CH_3_CN_3_), Cs and X = Cl, Br, I) have garnered significant attention, at first for their potential in photovoltaic applications^1–3^, and more recently for their remarkable performance in light-emitting and detection devices^4–10^. The exceptional optoelectronic properties of these materials originate from a successful combination of key physical characteristics, including long carrier lifetime, extended diffusion lengths, low exciton binding energy and high defect tolerance. By interchanging the components in the perovskite composition, a wide range of band gaps can be achieved, spanning from ultraviolet to infrared, while their electrical properties can be finely tuned from insulating to metallic^11^.

The successful applications of halide perovskites in direct X-ray detection^12–14^, raised expectations regarding their potential role in detecting ionising radiation which relies on indirect scintillation method^15–19^. The potential of metal-organic halide perovskites as scintillators has also been explored, with initial studies revealing extremely fast (< 1 ns) and bright scintillations at low temperatures^20,21^. In particular, the light yield of MAPbBr_3_ was estimated to be 116,000 ± 23,000 ph/MeV at 8 K approaching the theoretical limit. These findings have motivated further investigation into the scintillation properties of metal-organic bromides, chlorides, and mixed compounds^22–24^. Recent promising results reported by Xu at al^24^ and Mahato et al.^25^, who measured improvement of light yield at room temperature emphasises the importance of continued research into this family of materials as they are highly relevant for next-generation materials in medical diagnostics.

It is important to note that initial studies of scintillation properties were conducted on crystals less then millimetre in size. However, the detection efficiency for hard X-rays (> 100 keV) depends on both the composition, as the cross-section scales with Z^4–5^ (with Z being the atomic number), and thickness of the absorber. For halide perovskites, a ~ 1 mm thickness is required to fully absorb 100-keV X-rays^13^. Thus, it is crucial that high-quality scintillator crystals can be produced with a relatively large thickness and ideally with simple synthesis procedures. Here one of the key advantages of metal-organic halide perovskites is their ability to be fabricated at relative ease at low temperatures using solution-based techniques. For example, inverse temperature or anti-solvent vapor-assisted crystallization have enabled recently the growth of high-quality single perovskite crystals of a ~ 10 mm thickness at room temperature^26,27^. At the same time, when using perovskite crystals as scintillators a particular challenge arises from an increased re-absorption, which can significantly impact performance^28,29^. Thus, the potential of bulk single crystals of metal-organic perovskites for high-energy radiation detection requires further examination. In this work we explore the electronic structure of MAPbCl_3_ through computational modelling of the band structure and by reflectivity spectral analysis. Additionally, we investigate how temperature variations influence the dynamics of radiative decay of elementary excitations in the material when exposed to high-energy X-ray radiation.

## Experiment

**Fig. 1:**
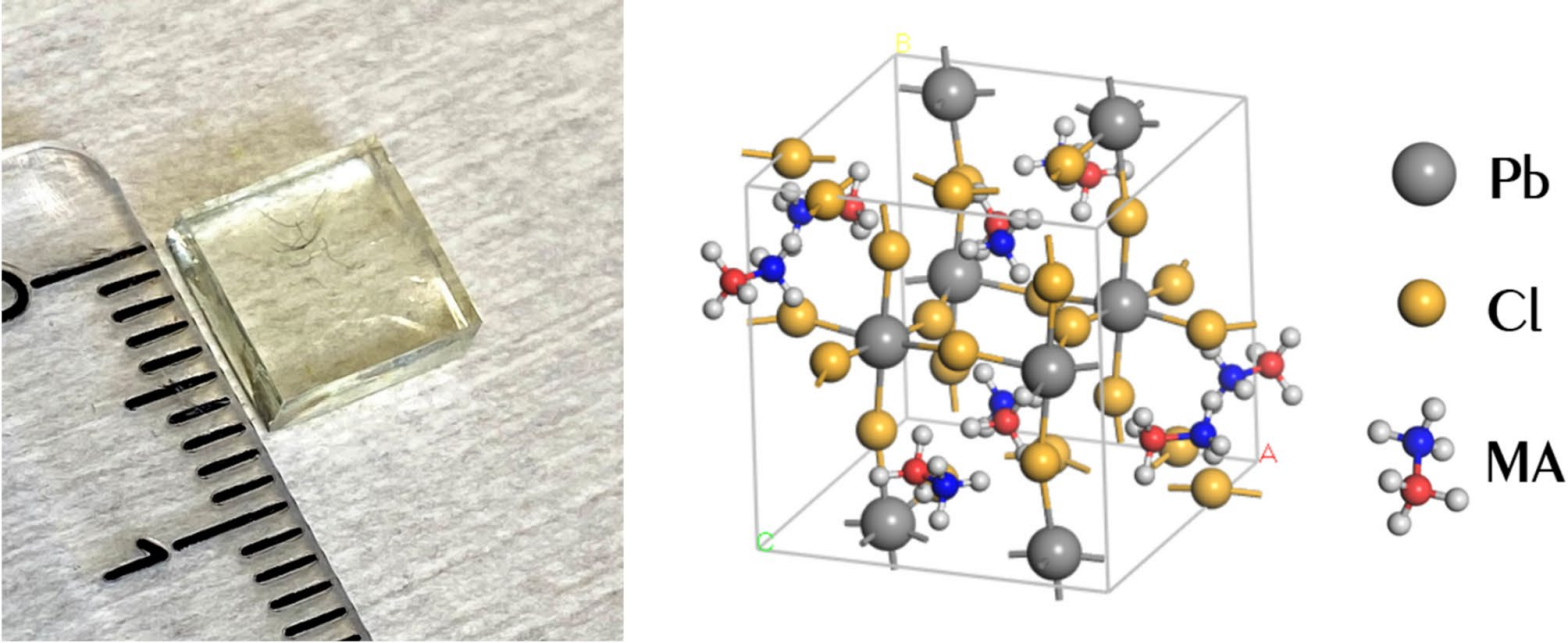
Left – photo of single crystal of MAPbCl_3_ with size 7 × 7 × 2 mm^3^. Right - the crystal structure of the orthorhombic phase (Pnma group) of MAPbCl_3_ where MA is methylammonium (CH_3_NH_3_).

For X-ray luminescence measurements the polished sample of MAPbCl_3_ crystal used in this study (see Fig. 1 left) was placed into a closed-cycle He cryostat. The experimental setup included a DE-202 A cryocooler (Advanced Research Systems) and Cryocon 32 temperature controller (Cryogenic Control Systems Inc.). The luminescence was excited by a URS-55 A X-ray tube with a Сu-anticathode tube operating at 55 kV and 10 mA. The luminescence spectra were measured at 45° to the incident radiation using an MDR-12 monochromator (5 nm spectral resolution) and photomultiplier module Hamamatsu H9305 sensitive in the wavelength range of 200–700 nm.

The decay curves of the crystals were measured at the Diamond Light Source synchrotron using 14 keV monochromatic X-ray pulses with a full width at half maximum (FWHM) of Δt = 60 ps. The crystal was placed in a continuous-flow He-cryostat (Oxford Instruments) with a Mylar window that is transparent to X-ray radiation. The sample temperature was monitored with a Si-diode sensor and stabilised using a PID controller. The X-ray beam was directed at the sample positioned at 45° to the incoming radiation. The luminescence from the illuminated area of 2 × 2 mm^2^ was collected in reflection mode at 45° through a quartz window. Emission was detected using an ID100 single photon counting detector sensitive over a 400–900 nm spectral range and a PICO Harp 300 time-correlated single photon counting module.

The high-resolution (1 nm) photoluminescence (PL) and the photoluminescence excitation (PLE) spectra were measured using a Horiba/Jobin-Yvon Fluorolog-3 spectrofluorometer with a 450 W continuous spectrum xenon lamp for excitation. The emission was detected by a Hamamatsu R928P photomultiplier operating in a photon counting mode. The measurements at low temperature were carried out in a Janis continuous-flow liquid helium cryostat. The reflectivity spectra in the UV range were recorded using the same Fluorolog-3 spectrofluorometer and a Horiba Quanta-phi integrating sphere. The reflectivity spectra in VUV range (3.7–11 eV) were studied at the PETRA III P66 beamline of the Deutsches Elektronen-Synchrotron DESY (Hamburg, Germany) using synchrotron excitation and a McPherson primary normal incidence monochromator. The angle between the normal to the sample surface and the incident photon beam was 17.5°. The reflected light excited sodium salicylate deposited onto a quartz window, and then luminescence of sodium salicylate was detected by a PMT R6358 photomultiplier and corrected on the spectral distribution of the exciting photon energy.

The electronic properties of MAPbCl_3_ in low-temperature orthorhombic phase with space group *Pnma* were studied using DFT calculations, as implemented in CASTEP^30^. The exchange-correlation functional was selected using the Perdew-Burke-Ernzerhof generalized gradient approximation modified for solids (GGA-PBEsol). The Brillouin zone (BZ) of MAPbCl_3_ was sampled using a 4 × 4 × 3 Monkhorst-Pack grid. To model the electron-ion interaction, ultra-soft pseudopotentials were employed, describing the electronic configurations as follows: 2s^2^2p^2^ for the C atom, 2s^2^2p^2^ for the N atom, 1s^1^ for the H atom, 5d^10^6s^2^6p^2^ for the Pb atom, and 4s^2^4p^5^ for the Cl atom. Geometry optimization, including lattice and bond length parameters, was fully relaxed using the Broyden–Fletcher–Goldfarb–Shannon (BFGS) minimizer^31^. The optimization procedure continued until the forces affecting each atom were less than 0.01 eV/Å. Given the presence of a heavy lead atom in the perovskite structure, the spin-orbit coupling (SOC) effect was incorporated into the DFT calculations to account for the strong relativistic effect expected between lead and halogen atoms.

## Results and discussion

### Band structure calculations and reflectivity spectra

Below 172 K, MAPbCl_3_ adopts a distorted perovskite structure with orthorhombic space group (*Pnma*)^32^ where the PbCl_6_ octahedra are tilted relative to the ideal perovskite geometry (see Fig. 1 right).

The arrangement of molecular ordering pattern induces a permanent structural distortion of the PbCl_6_ octahedra at low temperatures, resulting in an antiferroelectric striped pattern in the crystallographic *a*-direction^33^. Therefore, when modelling the crystal, we specifically chose this direction to be aligned with the MA molecules. Table 1 presents the calculated lattice parameters and lattice volume of unit cell for the orthorhombic phase of MAPbCl_3_.

The calculated results demonstrate strong agreement with the experimental data. This agreement was achieved by employing the PBEsol parametrization of the GGA, which was specifically developed to enhance the description of exchange interactions in solids. PBEsol provides improved structural parameters and total energy predictions for solids, addressing the limitations of the standard PBE, which tends to overestimate lattice parameters^34^. It is important to acknowledge that while PBEsol enhances the accuracy of conventional GGA functionals, previous studies have shown that hybrid functionals such as HSE06 or self-interaction corrected methods often provide a more reliable description of electronic properties, particularly in bandgap predictions^35,36^. Nevertheless, while PBEsol tends to underestimate the absolute value of the bandgap, this does not impact the further analysis of the results and still ensures an accurate reproduction of the energy band positions.

**Table 1:** Lattice parameters and volume for the orthorhombic phase of MAPbCl_3_, calculated using GGA-PBEsol compared with experimental results^37^.

MAPbCl_3_ orthorhombic Pnma	Lattice parameters			
	а (Å)	b (Å)	c (Å)	V (Å^3^)
Calculated	11.103	11.366	11.243	1418.95
Experimental	11.193	11.347	11.287	1432.5

Next, we calculated the band energy diagram which provides detailed insight into the electronic structure of MAPbCl_3_. The band energy diagrams for the orthorhombic crystal phase of the crystal are plotted along the high-symmetry points of the first Brillouin zone: Г→Z→T→Y→S→X→U→R, with the Fermi level set at 0 eV corresponding to the top of the valence band (VB).

**Fig. 2:**
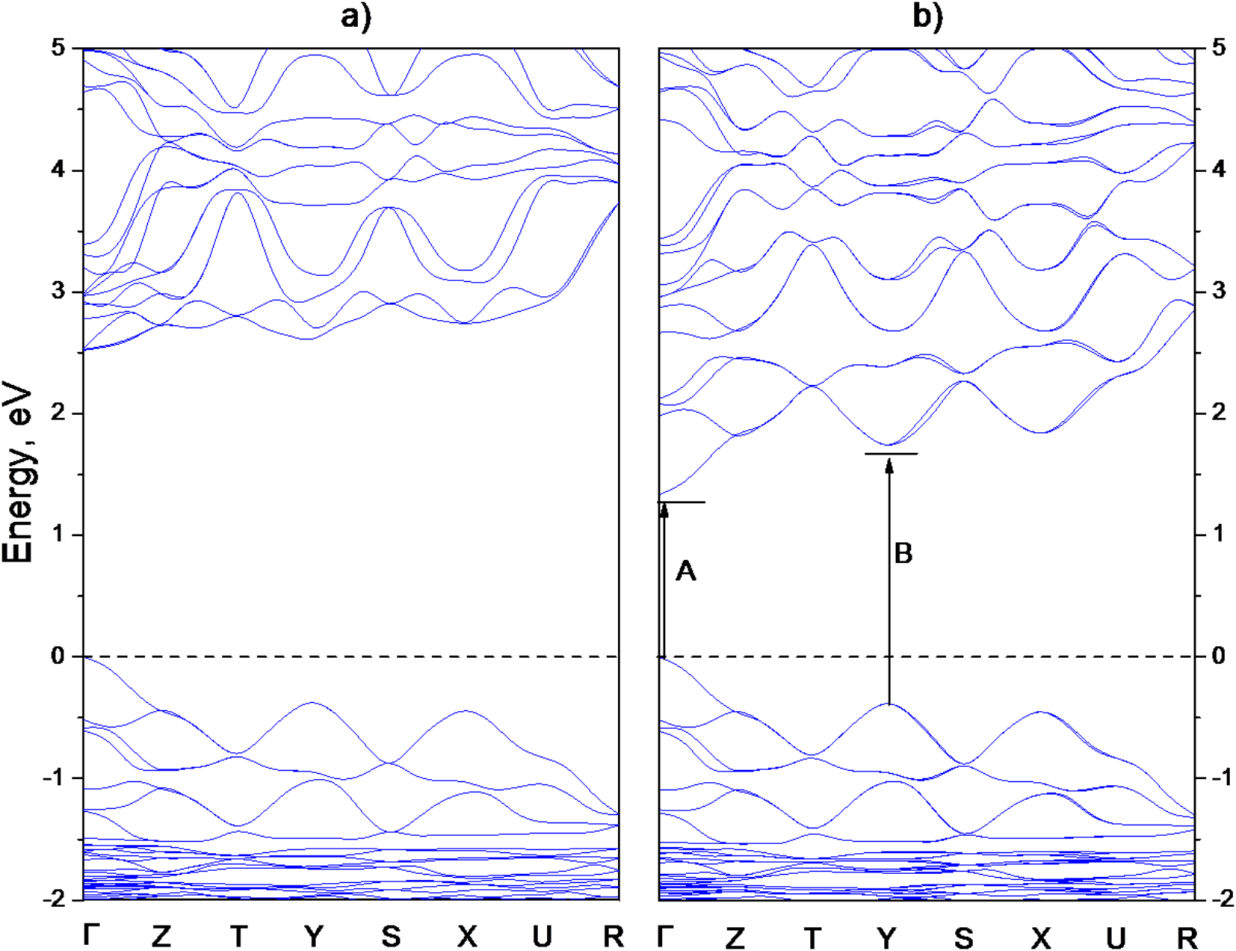
The band energy structure obtained using GGA-PBEsol functionals without (**a**) and with (**b**) Spin-orbit coupling (SOC) for orthorhombic MAPbCl_3_. The Fermi level indicates a dashed line at 0 eV. A and B mark the excitonic transitions observed in reflectivity.

The band energy diagrams for the low-temperature orthorhombic phase of MAPbCl_3_ perovskite reveal that both the top of the valence band (VB) and the bottom of the conduction band (CB) are located at the Γ point (see Fig. 2a), confirming the formation of a direct band gap. The calculations yielded a band gap of 2.52 eV, which is 0.6 eV lower than the experimentally measured value^38^. To more accurately capture the influence of the heavy Pb atom on the electronic structure of MAPbCl_3_, additional calculations were performed with the inclusion of the spin-orbit coupling (SOC) effect. Incorporating SOC significantly alters the band dispersions, which is crucial for interpreting the material optoelectronic properties^39^. The inclusion of SOC results in a narrowed band gap of 1.33 eV (Fig. 2b). This underestimation of the band gap is a known feature of SOC-inclusive calculations, as SOC induces a pronounced splitting of the CB in Pb-based perovskites^40^. The calculated band gap values, both with and without SOC, are consistent with previous theoretical estimates for the low-temperature phase of MAPbCl_3_, which reported gaps of 2.3 eV and 1.2 eV, respectively^41,42^. Despite the inherent limitations of DFT ground-state calculations, particularly their tendency to underestimate certain properties such as bandgap values, the derived conclusions about energy band dispersions and their symmetries remain robust. These results offer a reliable basis for construction of accurate semi-empirical Hamiltonians, especially in scenarios demanding precise descriptions of Bloch states and selection rules.

**Fig. 3:**
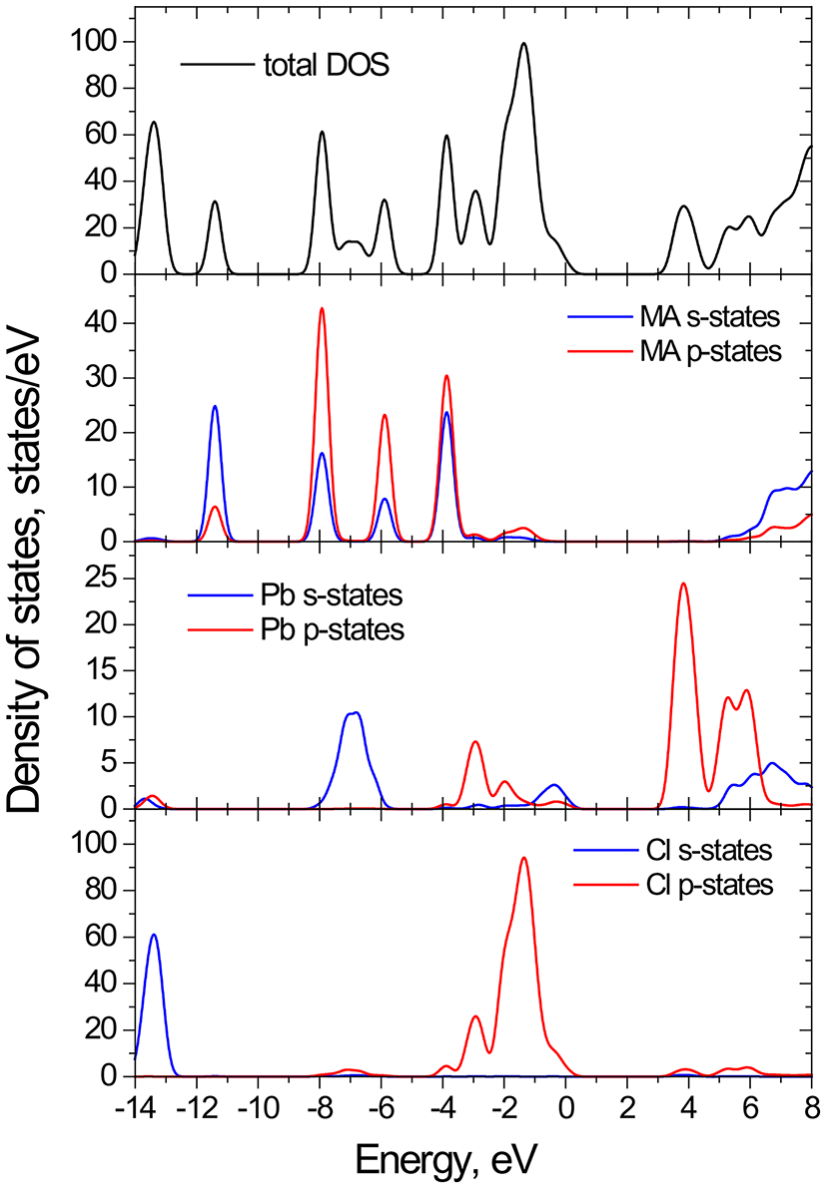
The DOS and PDOS calculated using GGA-PBEsol functional for orthorhombic MAPbCl_3_.

The nature of the valence band (VB) top and conduction band (CB) bottom in MAPbCl_3_ can be elucidated through first-principles calculations using density functional theory (DFT). The density of states (DOS) and projected density of states (PDOS) were computed over the energy range from − 14 to 9 eV, which can be broadly divided into three distinct regions corresponding to the bands shown in Fig. 3. The first region, spanning approximately from − 14 eV to −3 eV, corresponds to deep states within the VB. The second region, from − 3 eV to 0.4 eV, includes states that significantly contribute to the VB near the Fermi level. The third region, ranging from 2.9 eV to 6.8 eV, represents the upper portion of the CB. These calculations provide detailed insight into the electronic structure of MAPbCl_3_, highlighting the specific energy contributions to the VB and CB, and helping to understand the optoelectronic properties. As follow from these results most of chlorine s-states form deep energy levels around − 14 eV and have minimal impact on the conduction band (CB) or valence band (VB). Within the energy range of −11 to −3.5 eV, clusters of energetic s- and p-states from the methylammonium cation are observed, alongside hybridized s-states from lead. In MAPbCl_3_, the upper valence band is primarily composed of halide p orbitals, with minor anti-bonding contributions from Pb 6s² orbitals, while the conduction band is dominated by Pb 6p orbitals^41,43–45^. Although the molecular cation MA does not directly contribute to the electronic band edges^42^, it influences the crystal structure and indirectly affects the band gap.

MAPbCl_3_ is a direct band gap semiconductor exhibiting in high symmetry cubic phase the global extremum at the R point of the BZ and a secondary extremum at the M point. Modelling suggests the possibility of optically allowed transitions at both the R and M points, a phenomenon referred to as multiband gap absorption^46^. It is pertinent to remark that the electronic structure of the low-temperature orthorhombic phase has its origin in the cubic phase structure^47^; therefore in further discussion the notations of critical points from the highest symmetry phase are used for consistency. The strong spin-orbit coupling characteristic of materials containing heavy lead atoms leads to the formation of split states within the conduction band, further influencing the electronic properties of the material.

**Fig. 4:**
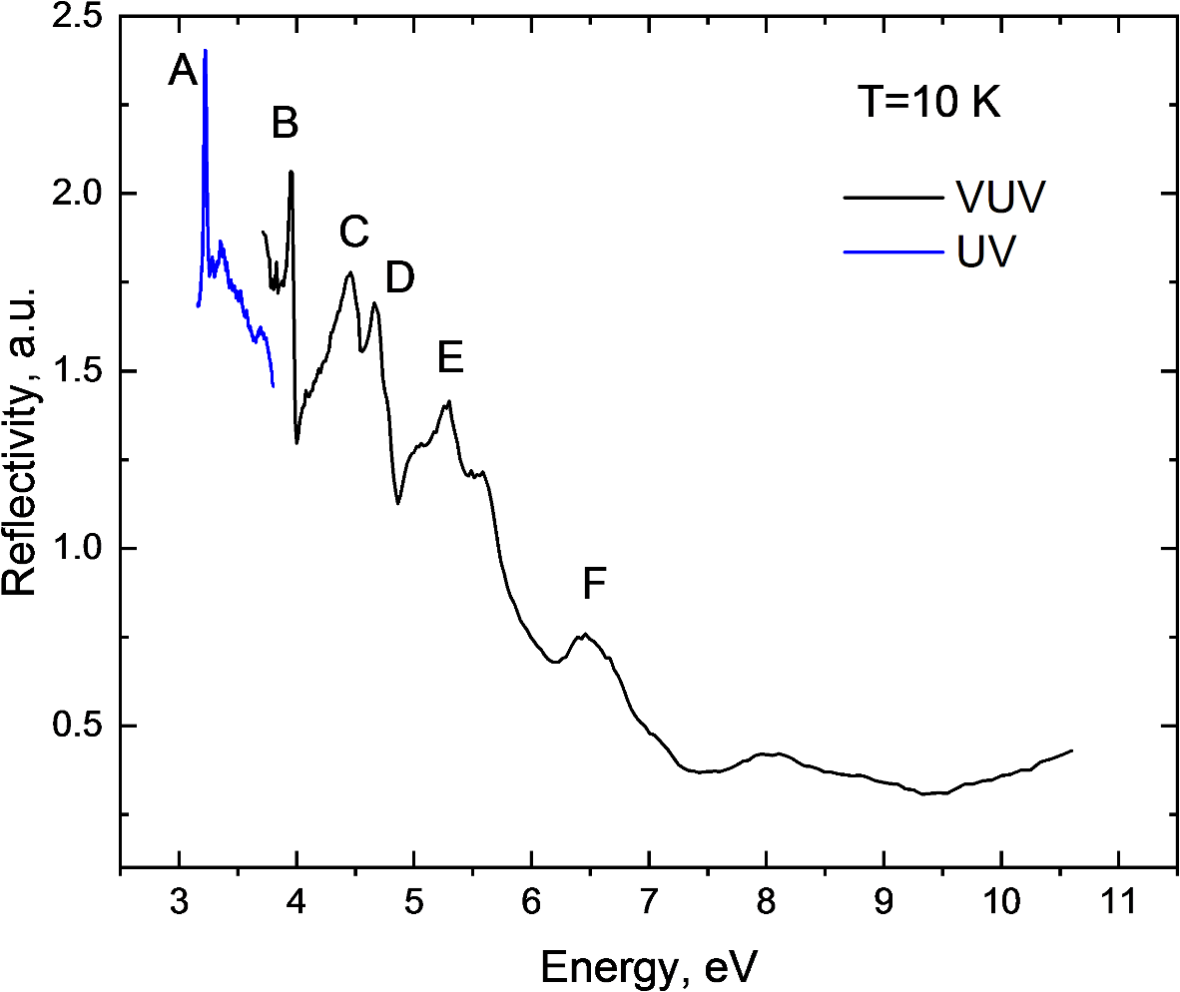
Reflectivity spectra of MAPbCl_3_ at 10 K. The spectra in VUV (black) and UV (blue) range are shown separately as they are measured using different experimental setups.

It should be noted that the calculations do not account for electron-hole interactions and, therefore, cannot capture exciton effects, manifested as sharp peaks in different regions of the absorption and reflection spectra of the crystal. This limitation has significant implications for the photo physics of perovskites, as exciton effects are crucial for enabling efficient light emission and the generation of electron-hole pairs across a broader range of excitation energies^48,49^. Despite this limitation, the calculated band structure of MAPbCl_3_ still provides valuable insights, allowing for the assignment of key features observed in the crystal reflectivity spectrum, as shown in Fig. 4. The reflectivity spectrum exhibits a clear excitonic transition, labelled as peak A, at 3.22 eV. This is followed by a second sharp peak, marked as B, at 3.94 eV, with subsequent peaks C, D, and E appearing at 4.44, 4.66, and 5.36 eV, respectively.

The position of the peak A aligns with recent reports for MAPbCl_3_^50^. The excitonic character of the first sharp peak, observed at 3.22 eV in the reflectivity spectra of APbCl_3_ crystals, has been well-established and recognized for some time^51–53^. The excitonic nature of the first peak in the reflection spectra has also been confirmed for other metal-halide counterparts MAPbX_3_ with X = Br, I^50,54^. Interestingly, optical spectra measurements beyond the first peak are more common for inorganic perovskites^51–53,55–60^, in contrast to MAPbX_3_. Existing data primarily come from spectroscopic ellipsometry studies of thin films^44,45^. Thus, it is sensible to analyse the reflectivity of MAPbCl_3_ using as a guidance prior data on the reflectivity of CsPbCl_3_. Following this approach, the sharp peak B at 3.94 eV can be attributed to the optically allowed second exciton transition at the M point of BZ. Observation of such transition is consistent with the concept of multiband structure of metal-halide perovskite crystals discussed in^46^. The subsequent peaks, labelled C and D, are associated with transitions from the uppermost valence band to the upper regions of the conduction band at the R and M points, respectively. Peak E is likely due to transitions from the lower part of the valence band to various parts of the conduction band, influenced by the spin-orbit splitting of the Pb 6p states. Transitions involving the MA cation begin above 6 eV, resulting in band F. As photon energy increases, the involvement of additional states in the transitions increases, which can lead to a smearing of spectral features. This complexity introduces certain level of ambiguity in the interpretation of the reflectivity spectra in this higher energy range.

### X-ray luminescence spectra with temperature

**Fig. 5:**
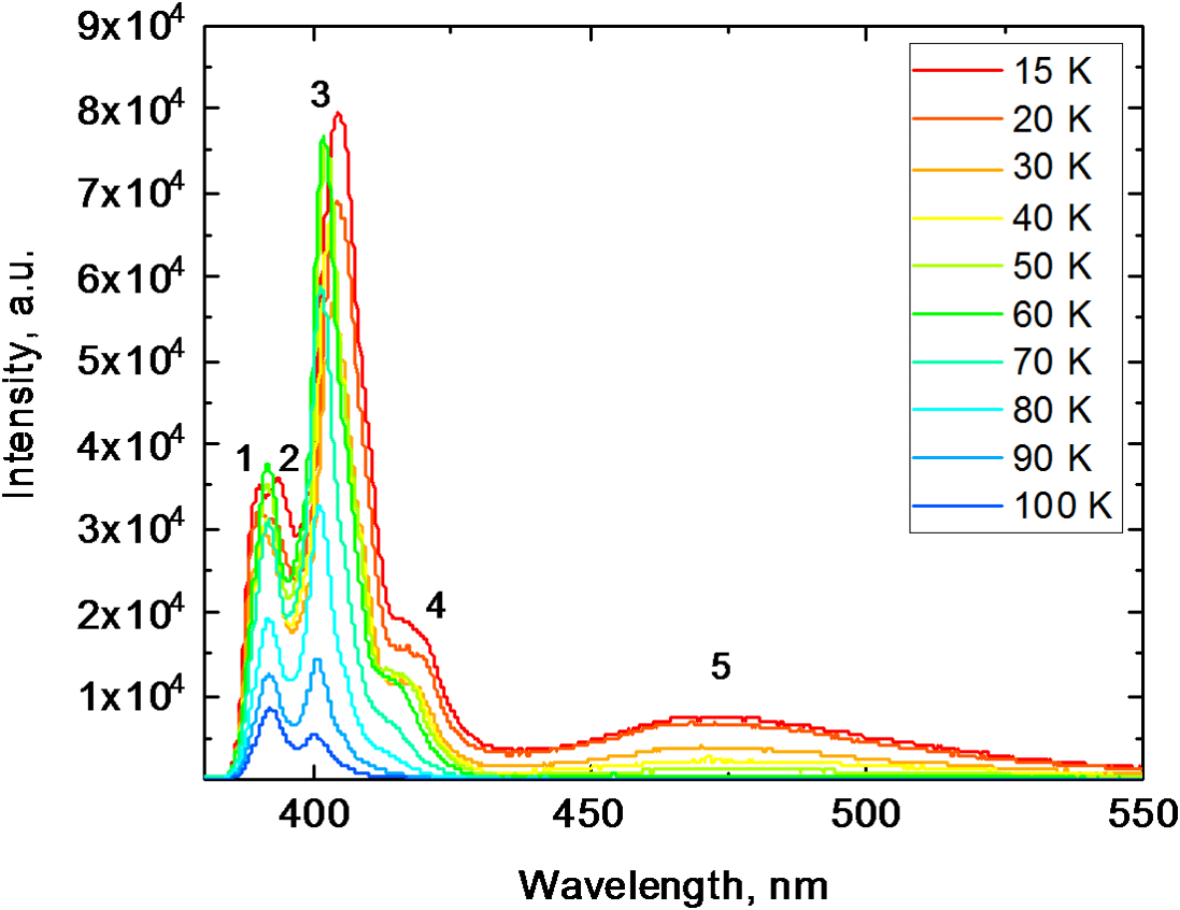
X-ray luminescence spectra of a MAPbCl_3_ crystal measured at different temperatures.

Previous studies of X-ray luminescence in MAPbCl_3_ crystals have shown significant thermal quenching, with peak intensity decreasing by two orders of magnitude when the temperature exceeds 150 K^22,61^. The X-ray luminescence spectra for the MAPbCl_3_ crystal measured in this work exhibit a similar pattern as illustrated in Fig. 5. At 15 K, the crystal shows distinct emission peaks at 389 nm (1), 392 nm (2), and 404 nm (3), along with a broad shoulder at 418 nm (4). Additionally, a weaker broad emission band is observed at 470 nm (5), extending from 440 to 550 nm.

As temperature increases, the spectra undergo notable changes: peaks 1 and 2 merge into a single band with a maximum at 391 nm. Peak 3 gradually shifts to 400 nm, while shoulder 4 and the broad band emission 5 disappear as the temperature rises above 70 K. With further temperature increase, the amplitudes of the remaining peaks at 391 and 400 nm continue to decrease due to thermal quenching. At 100 K, the luminescence intensity of MAPbCl_3_ is reduced by an order of magnitude compared to the value at 15 K.

**Fig. 6:**
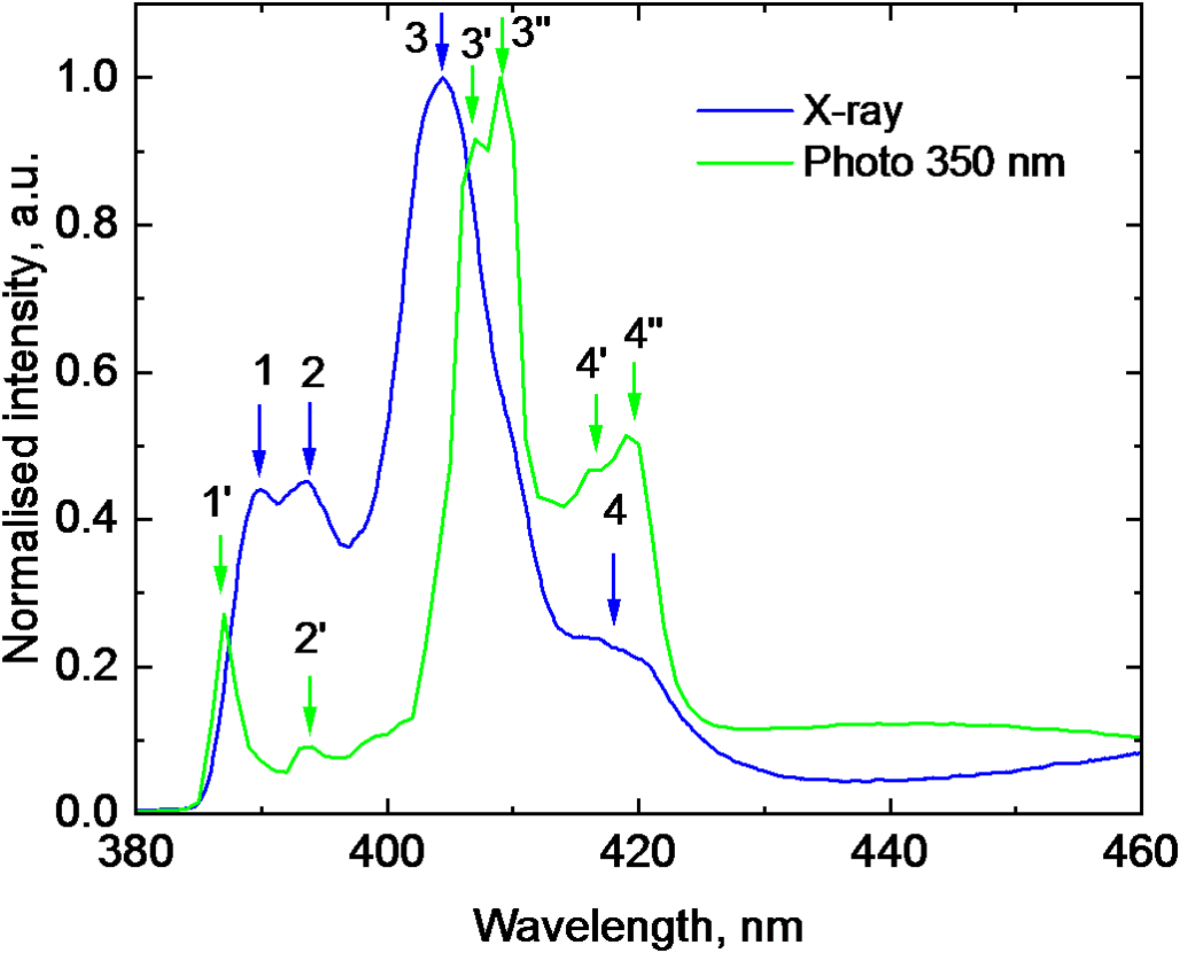
Luminescence spectra of a MAPbCl_3_ crystal measured at excitation with X-rays and photons 350 nm (T = 15 K). The assignments of specific peaks marked by numbers is provided in the text.

There is relatively large body of published results on the luminescence properties of organic-inorganic perovskites which are commonly used for interpretation and assignment of the main features observed in the X-ray luminescence spectra. This, however, requires caution because of a strong near-edge absorption and excitation density effects that can cause significant shape alteration of the observed spectra. Specifically absorption can cause red shift in the emission peaks^62^ while variation of excitation density can lead to changes in the intensity distribution of these peaks^28,29^. To rationalise the interpretation of X-ray spectra, we measured the luminescence spectrum of MAPbCl_3_ at photoexcitation at 350 nm. The X-ray and photoluminescence spectra recorded at T = 15 K are compared in Fig. 6.

In direct semiconductors the highest energy sharp peak in the luminescence spectra is typically associated with the radiative decay of free excitons^48^. This assignment is also commonly applied to the first peak observed at 387 nm (denoted as peak 1′) in the photoluminescence spectra of MAPbCl_3_^22,24,38,50^. Due to the short penetration depth of photons of 350 nm (< 10^− 8^ m), the emission from free excitons originates from the thin surface layer of the crystal.

In contrast, X-ray excitation creates the electronic excitations within a much thicker layer of the crystal, on the order of 10^− 4^–10^− 3^ m, depending on the energy of X-rays^63^. This difference in excitation depth leads to a redshift in the emission of free excitons from the bulk of the crystal. Therefore, with a relatively high degree of certainty, the peak at 389 nm (peak 1) observed in the X-ray luminescence spectra can be attributed to the radiative decay of free excitons, consistent with findings from previous studies^22^. Nonetheless, it is worth mentioning a less likely, yet still possible, explanation for the observed spectral changes—structural modifications in the excited states induced by high-energy X-rays.

The sharp peaks between 390 and 430 nm, labelled 2′, 3′, 3′′, 4, and 4′′ in the photoluminescence spectra of MAPbCl_3_ crystals at low temperatures, are frequently reported in literature^24,38,50,64^. These peaks are believed to have an excitonic origin and are attributed to the radiative decay of excitons bound to traps and defects. Therefore, it is reasonable to extend this interpretation to the corresponding peaks 2, 3, and 4 observed in our X-ray luminescence spectra. It should be noted that due to the lower spectral resolution of our experimental setup, the structure of peaks 3 and 4 in the X-ray luminescence spectra remains unresolved. The broad low-energy band 5 observed in our X-ray luminescence spectra at 470 nm is tentatively attributed to the defect-related emission, similar to emission observed in other metal-organic halide perovskites^22,50,65^. The emission in this spectral range exhibits decay time (~ 10^− 6^ s) which is supporting the proposed assignment. The trapping may occur at halide vacancies, which are the predominant deep defects in the MAPbCl_3_ crystal predicted by theory^66^.

### Luminescence decay at X-ray excitation

**Fig. 7:**
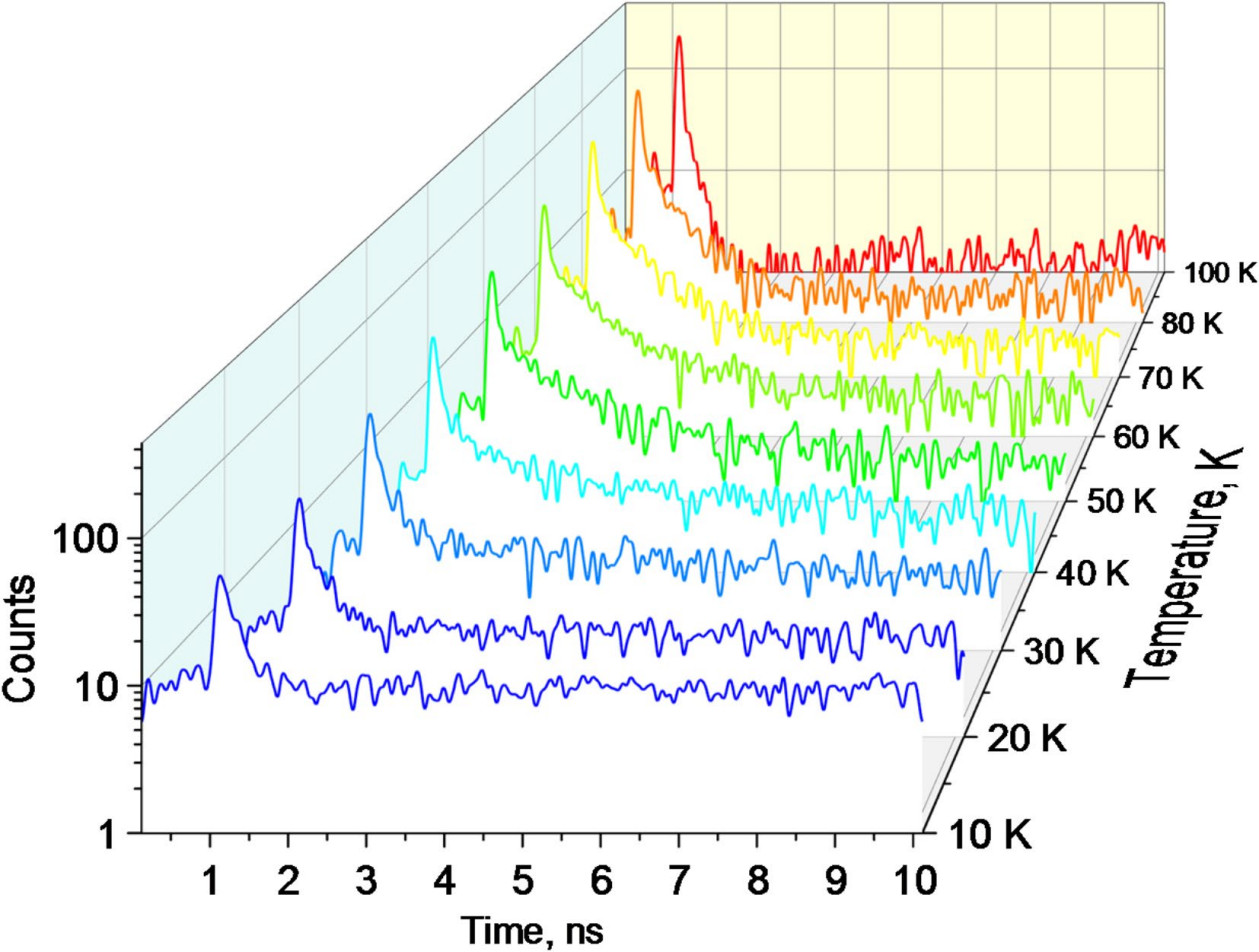
Decay curves of X-ray luminescence measured in the MAPbCl_3_ crystal at different temperatures. The luminescence is excited by X-ray pulses of synchrotron radiation at energy 14 keV.

To get further insight into the dynamic of emission processes in the crystal under study we investigated the kinetics of luminescence decay under pulsed X-ray excitation from a synchrotron source. The changes of the measured decay curves of MAPbCl_3_ crystals with temperature are displayed in Fig. 7. These decay curves, recorded in integral mode (capturing photons across the entire luminescence spectrum of the crystal), reveal complex kinetics resulting from various recombination processes and emission centres. Initially, the decay curves exhibit a rapid decrease in intensity following the excitation pulse. To accurately characterize the decay processes, the measured curves were subjected to deconvolution analysis^67^. This analysis allowed us to isolate and recover the intrinsic luminescence decay characteristics by accounting for the known instrumental response function (IRF).

**Fig. 8:**
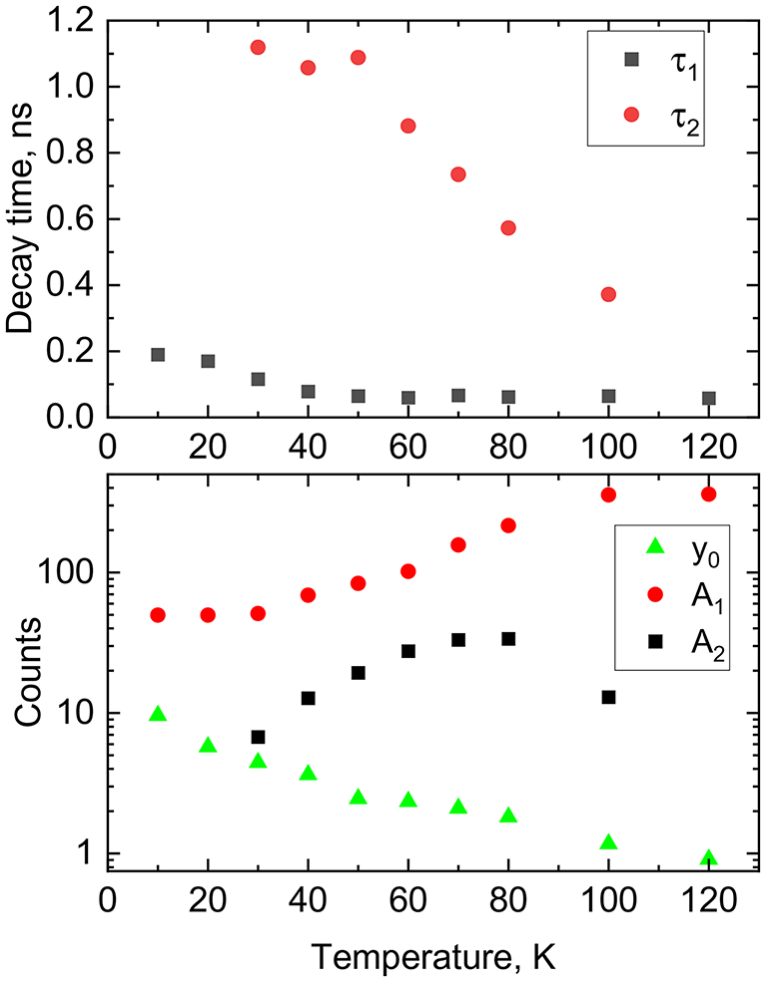
Temperature dependence of decay constants (top) and amplitudes with background (bottom) obtained from the fitting of the decay curves of MAPbCl_3_ by a sum of two exponential functions.

Following the deconvolution the decay curves were fitted using the sum of two exponentials:$f\left( t \right)={A_1}{\text{exp}}\left( { - t/{\tau _1}} \right)+{A_2}{\text{exp}}\left( { - t/{\tau _2}} \right)+{y_0}$ where ${A_{1,2}}$ and ${\tau _{1,2}}$ are the amplitudes and decay time constants of the two emission components, ${y_0}$ is a constant background. At very low temperatures, a single exponential function plus background was sufficient to fit the data. However, at temperatures above 20 K, a second exponential component was necessary to account for the slower decay process. The temperature-dependent decay time constants and amplitudes obtained from the fits are presented in Fig. 8.

The initial phase of the luminescence decay is characterized by a sub-nanosecond component with a decay time of approximately 0.2 ns at 10 K. This extremely rapid decay is indicative of excitonic processes occurring on a similar timescale, a feature that has been observed in other halide perovskites^25,28,54,68,69^. At elevated temperatures, a delayed decay component with a time constant of around 1 ns becomes evident. This longer-lived component is likely due to the radiative recombination of bound excitons released from shallow traps^69,70^. A very slow emission component with a decay time exceeding 10^− 7^ s, associated with the radiative recombination of electrons and holes from deeper traps, is not distinguishable in the measured decay curves at these timescale as it primarily contributes to the background. It is notable that the amplitudes of both decay components initially increase with temperature, suggesting that these recombination channels are enhanced by thermally stimulated processes. Specifically, the exciton channels are likely to be fed by the thermally induced release of trapped excitons, as indicated by the decrease in the amplitude of the background component with rising temperature. This behaviour is consistent with previous observations in MAPbI_3_ crystals, where changes in luminescence kinetics with temperature were attributed to the exchange of particles between different radiative decay channels^21^.

## Conclusion

In this study, we investigated the electronic structure of MAPbCl_3_ in its orthorhombic low-temperature phase through computer modelling of its band structure. The results of these DFT calculations were used to interpret key features of the crystal reflectivity spectra, particularly in the region of fundamental absorption (up to 11 eV), which has not been explored previously. Our analysis confirmed the presence of a sharp excitonic transition at 3.22 eV, corresponding to the R point of the BZ, as consistently reported in previous studies of MAPbCl_3_ reflectivity spectra. Additionally, we observed a second sharp peak at 3.94 eV, likely associated with an exciton transition at the M point of the BZ. The spectral features between 4 and 6 eV are attributed to transitions from the valence band, primarily formed by halide p-orbitals, to various regions of the conduction band, shaped by Pb 6p orbitals, at the R and M points. Transitions involving the valence states of the methylammonium anion were identified starting above 6 eV.

To explore how temperature variation affects the dynamics of radiative decay under high-energy irradiation, we measured the luminescence spectra and decay times of MAPbCl_3_ crystal down to 10 K, using steady-state and pulsed X-ray excitation, respectively. Our analysis revealed that at low temperatures, the luminescence is dominated by emissions from free and bound excitons in the 385–430 nm range, characterized by rapid decay times (≤ 1 ns) and significant thermal quenching. We also observed a weak broad emission band spanning 430–550 nm, tentatively attributed to the radiative decay of defects. These findings are consistent with previous results and enhance our understanding of the dynamic emission processes in MAPbCl_3_ crystals.

## Data Availability

Data generated during the current study are available from the corresponding author on reasonable request.
